# Parental imprisonment as a risk factor for cardiovascular and metabolic disease in adolescent and adult offspring: A prospective Australian birth cohort study

**DOI:** 10.1016/j.ssmph.2022.101107

**Published:** 2022-04-28

**Authors:** Michael E. Roettger, Brian Houle, Jake Najman, Tara R. McGee

**Affiliations:** a School of Demography, 146 Ellery Crescent, The Australian National University, Acton ACT, 2601, Australia; b MRC/Wits Rural Public Health and Health Transitions Research Unit (Agincourt), School of Public Health, Faculty of Health Sciences, University of the Witwatersrand, Johannesburg, South Africa; c School of Public Health, Public Health Building, The University of Queensland, Herston, 4006, Australia; d School of Criminology and Criminal Justice, Griffith University, 176 Messines Ridge Road, Mount Gravatt, QLD, 4122, Australia

**Keywords:** parental imprisonment, body mass index, systolic blood pressure, diastolic blood pressure, waist circumference, cardiometabolic risk, BMI, Body Mass Index, SBP, Systolic Blood Pressure, DBP, Diastolic Blood Pressure, AOR, Adjusted Odds Ratio, kg, kilograms, cm, centimeters, mmHG, millimeters of mercury, m, meters, CI, confidence interval

## Abstract

**Objectives:**

Parental imprisonment is linked with child health in later life. The present study provides the first prospective cohort analysis and non-U.S. based study examining parental imprisonment and cardiometabolic risk factors in adolescence and adulthood.

**Methods:**

The study followed 7,223 children born from live, singleton births from 1981 to 1984 in Brisbane, Australia. Data on parental imprisonment was collected at mother interview when the children were ages 5 and 14. Our sample analyzes offspring with biometric data collected by health professionals, including 3,794 at age 14, 2,136 at age 21, and 1,712 at age 30. Analyses used multivariate linear and logistic regression, and time-varying growth curve models.

**Results:**

Among female respondents, parental imprisonment at ages ≤5 was associated with higher body-mass index (BMI) at ages 14, 21, and 30; higher systolic blood pressure (SBP) and diastolic blood pressure (DBP) at age 30; and increased sedentary hours, larger waist circumference, and odds of a high-risk waist circumference at age 30. Parental imprisonment when the child was aged ≤14 was associated with increased BMI and SBP at age 30 for females. In growth-curve models, parental imprisonment when the child was aged ≤5 and ≤ 14 among females was linked with increased BMI; parental imprisonment when the child was aged ≤5 was associated with increased SBP and DBP. No significant associations were observed for males.

**Conclusions:**

Using prospective cohort data, our results support research showing that parental imprisonment, particularly in early childhood, is associated with increased BMI, blood pressure, sedentary hours, and waist circumference in females in early adulthood. These findings implicate parental imprisonment as a risk factor for cardiometabolic health issues in later life among females.

## Highlights

• First prospective study examining parental imprisonment and cardiovascular risk using non-U.S. data.
• Study analyzes outcomes for children at ages 14, 21, and 30.
• Outcomes include BMI, waist circumference, systemic and diabolic blood pressure, and sedentary behaviors.
• Cardiometabolic risk factors strongest for females experiencing parental imprisonment at or below age 5.
• Findings hold in both cross-sectional and time-varying models.

## 1 Introduction

A growing body of research has demonstrated that parental imprisonment is linked to a range of physical health risks and conditions for offspring in adolescence and adulthood, including asthma, sexually transmitted infections, obesity/body mass index (BMI) gain, increased allostatic load, and premature mortality risk (Jackson et al., 2021; Le et al., 2019; Lee et al., 2013; Niño & Cai, 2020; Turney, 2014; Van De Weijer et al., 2018). However, longitudinal studies linking parental imprisonment with health in later life remain rare, with a 2018 literature review finding only one U.S-based longitudinal study examining parental imprisonment and offspring physical health from adolescence into adulthood (Wildeman et al., 2018). Research examining parental imprisonment and offspring health is critical for policies and interventions which may address emerging health disparities related to parental imprisonment at later stages in the life course (Massoglia & Remster, 2019; Wildeman & Wang, 2017).

Research examining parental imprisonment and child physical health is largely limited to U.S.-based studies. Danish and Dutch studies have linked parental imprisonment with increased risk of infant and adult mortality (Van De Weijer et al., 2018; Wildeman et al., 2014), while one recent Australian study has reported an association between parental imprisonment with limited physical functioning among male offspring (van de Weijer et al., 2021). This is important given the rise of mass imprisonment in the U.S. beginning the 1970s (Clear & Frost, 2015); in contrast, Australian imprisonment rates in the 1980s and 1990s remained closer to Canada, New Zealand, England & Wales and other European countries (Leigh, 2020). Presently, one-in-three young adults ages 18–29 in the U.S. have experienced parental imprisonment of any length, while large sub-populations of Hispanic and African American children are 3 and 5-times more likely than white children to experience parental imprisonment for one-year or more in state or federal prison by age 14 (Enns et al., 2019; Sykes & Pettit, 2019, pp. 11–23). In contrast, other Western countries such as Australia maintain an imprisonment rate of 3–5 times lower than the U.S., with 4% of children in the general population in Australia experiencing parental imprisonment by age 16 (Dennison et al., 2013; Fair & Walmsley, 2021) Examining health outcomes associated with parental imprisonment in general populations outside of the U.S. context is thus critical for evaluating the generalizability of results, as the present study does using an Australian dataset.

Due to its linkage with 200 chronic diseases, disability and premature death, BMI and obesity are important measures for linking parental imprisonment with offspring health risks at later stages in the life course (Jastreboff et al., 2019). However, research has been limited. Using longitudinal data from a US cohort, Roettger & Boardman observed longitudinal associations between respondent reports of parental imprisonment and their increased BMI gain among females in adolescence and adulthood (Roettger & Boardman, 2012). Turney reported a cross-sectional association between parental imprisonment and overweight/obesity among children, which was mediated by factors associated with parental imprisonment such as poverty and parental absence (Turney, 2014). Among female African Americans in middle-adulthood, Lee and colleagues observed that imprisonment of a family member, including parental imprisonment, was associated with obesity and diabetes (Lee et al., 2014). Two cross-sectional studies have found null associations for paternal imprisonment, and a reduced risk of obesity for maternal or joint parental imprisonment in childhood and early adulthood (Branigan & Wildeman, 2019; Lee et al., 2013); these studies are important for helping to establish that the association between parental imprisonment and offspring obesity risk may vary by factors such biological sex, age, and stages in the life course.

Research on the long-term effects of parental imprisonment illustrate how parental imprisonment may biologically link with increased cardiometabolic risk. Parental imprisonment is associated with poor health behaviors; for example, parental imprisonment is linked with sleep problems and poor eating behaviors in childhood, placing children at higher risk for increased BMI and reduce cardiometabolic health in later life (Jackson & Vaughn, 2017; Jansen et al., 2018). Parental imprisonment is also linked with unmet childhood health care needs, including mental health care, which may place children at risk for obesity (Turney, 2017; Winning et al., 2015). In adolescence parental imprisonment is associated with early menarche, while increased allostatic load, reduced telomere length, and heighted levels of *C*-Reactive protein in females are found to occur in adulthood (Boch & Ford, 2015; Del Toro et al., 2021; Mitchell et al., 2017; Niño & Cai, 2020; Zhang et al., 2019). As broader research on the biological embedding of childhood adversities suggests, parental imprisonment may thus result in increased risk for disease (Danese & McEwen, 2012; Moore et al., 2020). The findings of increased *C*-Reactive Protein levels, an important biological measure of stress and inflammation associated with increased risk of early cardiometabolic health issues in females (Choi et al., 2013; Liu et al., 2017; Onat et al., 2008).

Research on parental imprisonment has also found complex associations with child health and illness. Parental imprisonment is commonly listed as an adverse childhood experience and stressor associated with subsequent mental and physical health issues in childhood, adolescence, and adulthood (Centers for Disease Control and Prevention, 2010; Finkelhor et al., 2015; Foster & Hagan, 2013; Turney, 2014). Longitudinal studies capturing change over time have shown that the effects of parental imprisonment are also compounded by related health-related adversities throughout the life course (Giordano et al., 2019; Murray et al., 2012; Roettger & Houle, 2021; Turney, 2014; Wildeman & Andersen, 2017). Children experiencing parental imprisonment are often exposed to multiple traumas and adversities such as parental substance use, poverty, child abuse/neglect, parental absence, social exclusion, and food insecurity (Giordano, 2010; Giordano & Copp, 2015; Giordano et al., 2019; Nesmith & Ruhland, 2008; Nichols et al., 2016). As adolescents and adults, children experiencing parental imprisonment often also have co-occurring adversities that include poorer educational outcomes, poorer health behaviors, mental illness, substance use issues, and higher rates of antisocial behavior and imprisonment; these patterns are typically intergenerational (Giordano et al., 2019; Heard-Garris et al., 2018; Jackson et al., 2021; Roettger et al., 2011; Roettger & Swisher, 2011; Turney & Goodsell, 2018; Wakefield & Wildeman, 2013). The factors can accumulate across the life course into health disparities and early risk of illness, as a component of more systemic intergenerational social exclusion and disadvantage (Foster & Hagan, 2015).

While the complexity of factors which link parental imprisonment to health must be acknowledged, it remains important to note that parental imprisonment itself is a risk factor for adverse health outcomes in later life. Given parental imprisonment may lead to factors such as poverty, family instability, and residential instability, parental imprisonment also serves as a proxy for an array of related traumas and adversities. Examining parental imprisonment and adverse childhood experiences, Jackson et al. (2021) found that parental imprisonment was an independent risk factor for poorer physical health; however, this association became insignificant for the subset of children also experiencing additional adversities after adjusting for the number of adverse childhood experiences. Roettger and Houle (2021) found that paternal imprisonment was linked to increased risk of STI infection in early adulthood, but also observed that a range of adversities mediated this association. Parental imprisonment has also been linked to increased risk of antisocial behavior in fixed-effect modelling and quasi-experimental research (Murray et al., 2012; Wildeman & Andersen, 2017). Even without controlling for underlying causal mechanisms, establishing how parental imprisonment may be linked with cardiometabolic disease risk in adulthood due to experienced related childhood adversities is critical for establishing associations beneficial future research and policy.

### 1.1 Contributions of current study

Using a prospective Australian cohort study, we examine the relationship between parental imprisonment in childhood and obesity-related risks at age 30 by addressing four limitations in the existing literature.

First, studies examining parental imprisonment BMI gain, obesity, and metabolic risk have relied on non-prospective U.S. samples (Wildeman et al., 2018). By examining these risks using Australian data, it is possible to see if these associations hold both internationally and in a Western country absent the context of mass imprisonment for the general population. The use of prospective cohort data is valuable in better establishing causal associations over time.

Second, existing studies have almost exclusively examined cross-sectional associations, which do not allow for measuring change over time to better establish temporal sequencing. Furthermore, no studies directly compare cross-sectional and longitudinal results using prospective cohort data to examine the consistency of findings (Wildeman et al., 2018; Wildeman & Lee, 2021). By using a prospective cohort, we can compare cross-sectional results with those examining how these associations vary over time to better evaluate the consistency of findings.

Third, recent research suggests that experiencing parental imprisonment early in the life course may have greater impact than in adolescence, though this finding has not been examined using prospective imprisonment data to examine physical health (Turney, 2021). The current data allows us to test if those reporting imprisonment through age 5 and through age 14 have different associations with cardiometabolic risk. This is important for determining if early childhood experiences of parental imprisonment lead to higher cardiometabolic risk, as is suggested by research linking early childhood poverty with adult cardiometabolic risk in women (Najman et al., 2020). The data also provide a clearer picture of the extent to which categorization of imprisonment at different periods in childhood may to differing results, given that Roettger and Boardman (2012) examined risk of imprisonment prior to age 18.

Finally, some research suggests differential effects of parental imprisonment on BMI-related measures for females compared to men (Lee et al., 2014; Roettger & Boardman, 2012). As noted earlier, research has suggested that females who experience parental imprisonment are more likely to experience early menarche and have higher levels of *C*-Reactive Protein (an indicator of chronic stress); these biological factors are linked with increased cardiometabolic risks in females in young adulthood, generally, and may theoretically underlie an association between parental imprisonment and cardiovascular risk for females who experience parental imprisonment in childhood.

## 2 Material and methods

### 2.1 Data

We use data from the Mater Hospital-University of Queensland Study on Pregnancy (MUSP). The MUSP is a cohort study of 7,223 mothers whose pregnancies resulted in live, singleton births from 1981 to 1984 in the obstetrics unit of the Mater-Misericordiae Hospital in Brisbane, Australia. Mother and child clinical and survey data have been collected at several follow-up waves until the children reached age 30. This study uses data collected from mothers prenatally and when their children were aged 5 and 14; and child data are from waves of data collected at ages 14, 21, and 30. Among the 7,223 children who were initially enrolled in the study, we examine subsets of respondents who had clinical biometric data completed at ages 14, 21, and 30 during physical assessments by a trained health professional. These numbers include 3,794 respondents at age 14, 2,136 respondents at age 21, and 1,712 respondents at age 30. Further details of the MUSP data are available in the MUSP cohort profiles and research publications (Najman et al., 2005, 2015).

The attrition in the sample is a potential issue for the representativeness of the data. As noted in two MUSP cohort profiles, early and later attrition through age 30 have not been found to substantially bias results for parent and child outcomes in the MUSP and the representativeness of the original Brisbane sample (Najman et al., 2005, 2015). Attrition analyses of the MUSP data for mothers found that having problems with the law was not associated with increased attrition, while ethnic minorities and those with lower SES were more likely be lost in the sample (Saiepour et al., 2019); to address this potential attrition bias, we control for family SES and ethnicity in the analysis. Attrition for biometric measures, such as blood pressure, height, and weight, has been limited due to collection of these measures at the Mater Misericordiae clinic, limiting access for those who may have moved outside of the Brisbane area (Das et al., 2020). However, this limitation for the biometric data has not been found to bias results using the age 21 and age 30 cohort data in research (Das et al., 2020; Najman et al., 2020).

Ethics approval was received from relevant committees at The University of Queensland and the Mater Misericordiae Hospital, South Brisbane, Australia for data collection. For the present study, we use deidentified secondary data exempt from Human Ethics approval.

To maintain confidentiality, data from the MUSP are not made publicly available. Data may be obtained from the University of Queensland through the study website at: https://social-science.uq.edu.au/mater-university-queensland-study-pregnancy?p=9#9.

### 2.2 Dependent variables

Body mass index (BMI, *kg/m*^*2*^). Based on measured height (meters) and weight (kilograms) during physical assessments at ages 14, 21, and 30. Normal body mass is in the range 18.5 ≤ BMI<25, overweight BMI is in range 25 ≤ BMI<30, and obesity is BMI≥30 (Conen et al., 2007).

Systolic Blood Pressure (SBP). SBP (mmHG) at ages 21 and 30 was measured during physical assessments. Two readings were taken 5 min apart when the respondents were sitting and at rest. The respondent's SBP was the average of these two readings (Das et al., 2020). For SBP, categories are optimal for readings less than120 mmHG, normal is 120–129 mmHG, high-normal is 130–139 mmHG, and hypertension is ≥ 140 mmHG (Conen et al., 2007).

Diastolic Blood Pressure (DBP). DBP (mmHG) at ages 21 and 30 was measured during physical assessments. Two readings were taken 5 min apart when the respondents were sitting and at rest. The respondent's DBP was the average of these two readings (Das et al., 2020). For DBP, optimal blood pressure is > 75 mmHG, normal is 74–84, high-normal is 85–89 mmHG, and hypertension is ≥ 90 mmHG (Conen et al., 2007).

Sedentary hours. Number of self-reported hours per day over the prior week spent watching TV or using a computer for non-work purposes at age 30. Increased sedentary time, such as TV viewing time, is linked to increased cardiovascular risk (Wijndaele et al., 2010).

Waist size. Self-reported waist size (centimetres) at age 30. To measure waist size, respondents were provided with a paper measuring tape and detailed instructions.

High-risk waist size. An indicator for respondent waist sizes measured at age 30 being ≥88 cm for females and ≥102 cm for men. These waist sizes are considered to be strongly associated with subsequent risk of cardiometabolic diseases (Klein et al., 2007).

### 2.3 Predictor variables

Parental imprisonment. When children were ages 5 and 14, biological mothers were asked if they or their current partner had been detained in prison. From these variables, two measures were constructed for (1) if the mother or current partner had ever been detained in prison before age 5 and (2) if the mother reported she or current partner had ever been detained in prison at age 5 and/or age 14 interviews. We note that ‘current partner’ to the biological mother may be either the biological father or a non-biological father.

Maternal education. Maternal education is based on the biological mother's self-reported educational level prior to birth. Using this measure, we construct an indicator for if the mother had not completed secondary school or had completed any tertiary educational studies. Mother's education is used to control for child socio-economic status.

Child birth weight. Measured birth weight in grams from obstetric reports. Child birth weight is a significant predictor of increased BMI and cardiometabolic disease risk in adulthood (Jornayvaz et al., 2016; Kinge, 2017).

Non-European ethnicity. Based on a constructed classification, an indicator for the child being of non-European ethnicity (i.e., of Asian and/or Indigenous Australian descent). This control allows us to adjust for potential ethnic variation in the sample.

Sex. Respondent sex at birth was classified from obstetric data. Sex at birth is both a control and a potential moderator of results in the sample.

Pregnancy status. At child ages 21 and 30, an indicator for female respondents who report being pregnant at the time of interview. To better compare results with Roettger and Boardman (2012), we include a control for whether or not the individual is pregnant to control for increased BMI related to pregnancy. We note that removing pregnant females from the sample did not substantively alter the results presented below.

### 2.4 Statistical analysis

We used multivariate OLS regression to estimate continuous outcomes at ages 14, 21, and 30. We used multivariate logistic regression to estimate the odds of having a high-risk waist size at age 30.

In addition, we used multivariate growth-curve models to estimate the association between parental imprisonment and time-varying measures of 1) BMI at ages 14, 21, and 30 and 2) systolic and DBP at ages 21 and 30. In doing so, we model the time-varying variations of these measures associated with parental imprisonment by using a random individual-level intercept. As Curran and colleagues note (Curran et al., 2010), this analysis allows us to make use of partial-data for individuals across waves and also determine if the associations observed at single waves hold as individuals progress through the life course. The estimation of change over time provides more robust findings holding across waves, relative to single-wave trends which may be influenced by single-wave attrition.

To examine sex differences in risk, we estimate results for (1) pooled sex, (2) males only, and (3) females only. We conducted analyses using Stata 15.1. In the statistical analyses from regression analyses presented below, we report the unstandardized beta coefficient or adjusted odds ratio, along with 95% confidence intervals.

## 3 Results

Descriptive statistics and associated p-values for variables used in the analyses are presented in Table 1. These variables are organized by mother's report of parental imprisonment (1) at offspring age ≤5 and (2) at age ≤14. Parental imprisonment at ages ≤5 is associated with significantly higher BMI at ages 21 (p < 0.001) and 30 (p < 0.000) and waist circumference (p < 0.05), while mothers are significantly less likely to have completed a tertiary education. Parental imprisonment at ages ≤14 is significantly associated with higher BMI (p < 0.05), while also being significantly associated with lower maternal educational attainment.

**Table 1:** Means and standard deviations for variables used in analysis, by parental history of imprisonment (1) prior to age 5 and (2) prior to age 14.

	(1) Parental Imprisonment age ≤5 years					(2) Parental Imprisonment age ≤14 years				
	Parental Imprisonment		No Parental imprisonment		p-value	Parental Imprisonment		No Parental imprisonment		p-value
	MEAN/%	SD	MEAN/%	SD		MEAN/%	SD	MEAN/%	SD	
Dependent Variables										
Age 14	[N = 44]		[N = 3378]			[N = 166]		[N = 3524]		
BMI	21.14	5.04	20.62	3.78	0.364	21.04	4.49	20.62	3.76	0.163

Age 21	[N = 41]		[N = 2277]			[N = 121]		[N = 2393]		
BMI	26.69	6.89	24.21	4.92	0.001	24.92	5.49	24.24	4.95	0.142
SBP	118.42	13.52	116.24	14.42	0.355	116.62	14.07	116.29	14.45	0.809
DBP	69.75	10.77	67.70	8.48	0.141	68.70	8.67	67.63	8.52	0.177

Age 30	[N = 19]		[N = 1532]			[N = 83]		[N = 1580]		
Sedentary hours	3.91	4.43	3.14	3.23	0.214	3.53	4.01	3.16	3.24	0.244
Waist circumference	97.45	18.98	90.29	14.93	0.038	92.67	15.38	90.24	14.90	0.153
High-risk waist circumference	57.90%		33.92%		0.028	42.17%		34.04%		0.132
BMI	32.08	9.75	27.03	6.02	<0.001	28.61	7.37	27.05	5.99	0.023
SBP	125.15	15.96	119.08	15.81	0.096	122.26	15.39	119.03	15.94	0.073
DBP	75.53	9.68	71.65	9.70	0.086	73.51	9.44	71.70	9.77	0.103

Controls										
Pregnancy age 14	0.00%		0.00%			0.00%		00.00%		
Pregnancy age 21	2.41%		2.81%		0.870	4.75%		2.74%		0.180
Pregnancy age 30	10.00%		6.87%		0.581	7.13%		6.88%		0.927
Respondent non-European ethnicity	13.82%		8.45%		0.061	11.22%		8.69%		0.141
Mother tertiary education	10.75%		18.88%		0.044	13.65%		18.62%		0.038
Mother non-secondary education	22.49%		16.92%		0.150	23.43%		17.01%		0.004
Respondent female	51.84%		48.16%		0.628	51.76%		48.24%		0.508
Child birth weight	3.36	1.56	3.40	0.52	0.495	3.35	0.29	3.40	0.52	0.180

In the results below, we note that the number of cases for respondents reporting parental imprisonment at age 30 is relatively small, so caution should be taken in the interpretation of these results. At age 30, the pattern of findings is used to interpret indicators of cardiometabolic risk in the sample.

Table 2 shows multivariate regression results for parental imprisonment and the outcome variables. For parental imprisonment at offspring age ≤5, results show significant associations for the pooled biological sex models, including increased BMI at ages 21 of 2.47 units (95% CI: 0.94, 4.00) and at age 30 of 4.51 units (95% CI: 1.44, 7.58). At age 30, the pooled sex models also showed increased SBP at 6.64 mmHG (95% CI: 0.89, 12.49), DBP at 4.14 mmHG (95% CI: 0.01, 8.32), waist circumference of 6.99 cm (95% CI: 0.51, 13.47), and 2.64 higher odds (95% CI: 1.04, 6.69) of having a high-risk waist circumference.

**Table 2:** Multivariate Regression Coefficients and 95% Confidence Intervals for of Parental Imprisonment on Cardiometabolic Risk Factors, by respondent age, timing of parental imprisonment, and respondent biological sex.

		BMI	SBP	DBP	Sedentary Hours	Waist Circumference	High Risk Waist Circumference
**Age 14**							
Parental imprisonment, ≤5 years							
Pooled Sex	Coef.	0.49					
	95% CI	-0.61, 1.52					
Males, only	Coef.	-0.96					
	95% CI	-2.52, 0.60					
Females, Only	Coef.	1.71*					
	95% CI	0.14, 3.28					
Parental imprisonment, ≤14 years							
Pooled Sex	Coef.	0.40					
	95% CI	-0.19, 0.98					
Males, only	Coef.	0.10					
	95% CI	-0.70, 0.91					
Females, Only	Coef.	0.72					
	95% CI	-0.14, 1.58					

**Age 21**							
Parental imprisonment, ≤5 years							
Pooled Sex	Coef.	2.47**	1.81	2.01			
	95% CI	0.94, 4.00	-1.92, 5.56	-0.65, 4.66			
Males, only	Coef.	0.53	-0.89	1.10			
	95% CI	-1.42, 2.50	-6.33, 4.54	-2.50, 4.71			
Females, Only	Coef.	4.33***	4.58	2.96			
	95% CI	1.99, 6.67	-0.56, 9.73	-9.94, 6.87			
Parental imprisonment, ≤14 years							
Pooled Sex	Coef.	0.69	-0.07	1.09			
	95% CI	-0.21, 1.60	-2.20, 2.05	-0.42, 2.60			
Males, only	Coef.	0.17	-0.81	1.20			
	95% CI	-0.97, 1.30	-3.87, 2.24	-0.84, 3.25			
Females, Only	Coef.	1.26	0.82	1.01			
	95% CI	-0.16, 2.70	-2.15, 3.78	-1.24, 3.24			

**Age 30**							
Parental imprisonment, ≤5 years							
Pooled Sex	Coef./AOR	4.51**	6.64*	4.14*	1.35	6.99*	2.64*
	95% CI	1.44, 7.58	0.89, 12.40	0.01, 8.32	-0.29, 2.98	0.51, 13.47	1.04, 6.69
Males, only	Coef./AOR	1.33	2.86	1.54	-1.43	-1.73	1.60
	95% CI	-2.71, 5.42	-6.23, 11.97	-4.99, 8.08	-4.32, 1.45	-11.28, 7.81	0.37, 6.90
Females, Only	Coef./AOR	6.62**	9.24*	5.87*	2.61**	13.60**	4.14*
	95% CI	2.28,10.96	1.73, 16.76	0.39, 11.34	0.62, 4.59	4.77, 22.41	1.08, 15.79
Parental imprisonment, ≤14 years							
Pooled Sex	Coef./AOR	1.49*	2.71	1.46	0.13	2.37	1.36
	95% CI	0.04, 2.94	-0.19, 5.61	-0.64, 3.57	-0.66, 0.91	-0.84, 5.59	0.85, 2,17
Males, only	Coef./AOR	0.80	1.21	0.27	0.33	0.02	1.33
	95% CI	-1.09, 2.69	-3.24, 5.66	-2.93, 3.47	-0.98, 1.63	-4.61, 4.66	0.64, 2.80
Females, Only	Coef./AOR	1.89*	3.85*	2.37	-0.03	4.21	1.38
	95% CI	0.02, 2.94	0.02, 7.63	-0.43, 5.17	-1.02, 0.95	-0.25, 8.67	0.75, 2.53

Notes: Significance: *p<0.05 **p<0.01 ***p<0.001. Abbreviations: Coef.=Unstandardized Beta coefficient, AOR=Adjusted Odds Ratio, 95% CI=95% Confidence Interval, BMI=Body Mass Index, SBP=Systolic Blood Pressure, DBP=Diastolic Blood Pressure. Unless indicated otherwise, results are derived from OLS regression models. All models contain controls for non-European ethnicity, maternal education, and child birth weight. Pooled models contain controls for sex at birth and pregnancy status for females, while models for female respondents contain controls for pregnancy status. Blood-pressure models contain an additional control for use of blood pressure medications in year prior to interview.

However, when examining results by biological sex, significant associations are found only among female offspring. For females, parental imprisonment was associated with an increased BMI of 1.71 units (95% CI: 0.14, 3.28) at ages 14, 4.33 units (95% CI: 1.99, 6.67) at age 21, and 6.62 units (95% CI: 2.28, 10.92) at age 30. Parental imprisonment was associated with 2.61 h (95% CI: 0.62, 4.59) of increased sedentary behaviors at age 30, increased waist size of 13.60 cm (95% CI: 4.77, 22.41), and increased odds of 4.14 (95% CI: 1.08, 15.79) of a waist size >88 cm at age 30. Increased SBP, at 9.24 mmHG (95% CI: 1.73, 16.76) and DBP, at 5.87 mmHG (95% CI: 0.39, 11.34), were also associated with parental imprisonment at age 30. No significant results were observed for male respondents.

For parental imprisonment at offspring age ≤14, results show significant associations for the pooled biological sex models for BMI at 1.49 units (0.04, 2.94). Similarly to age ≤14, parental imprisonment among females was significantly associated with an increased BMI of 1.89 units (95% CI: 0.02, 2.94) and SBP of 3.84 mmHG at age 30 (95% CI: 0.02, 7.63). No significant associations were observed for male.

**Fig. 1:**
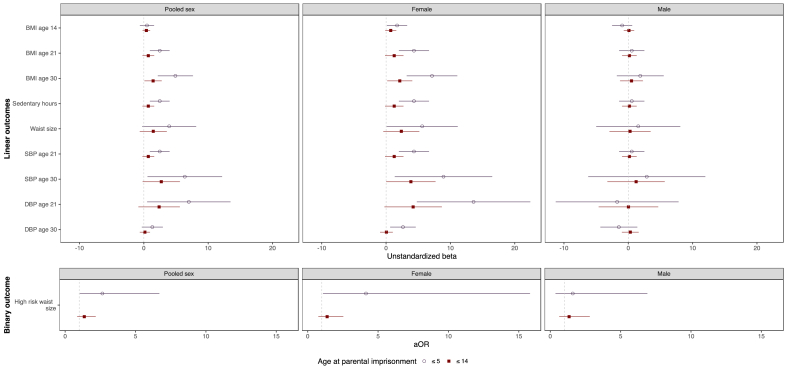
Estimated regression coefficients and 95% confidence intervals for outcomes associated with parental imprisonment at ages ≤5 and ages ≤14, by respondent sex. Plotted results are based multivariate OLS and logistic regression results presented in Table 2. Abbreviations: BMI=Body Mass Index, SBP=Systolic Blood Pressure, DBP = Diastolic Blood Pressure. ≤5 denotes parental imprisonment prior to age 5. ≤14 denotes parental imprisonment reported by mothers prior to age 14.

A summary of findings from Table 2 for pooled sex, along with separate results for male and female offspring are shown in Fig. 1. When pattern of significant associations for the pooled sex models are examined by gender, the plots show the substantive pattern of variation in statistical significance by respondent's biological sex described above. For female offspring, parental imprisonment is associated with a number of factors indicative of increased cardiometabolic risk, particularly for parental imprisonment experienced among females in early childhood (at ages of 5 or less). However, no significant associations were observed for males.

Table 3 shows results for individual growth curve models for BMI and SBP and DBP. At both ages ≤5 and ages ≤14, pooled sex models show a significant association with increased BMI at 1.37 units (95% CI: 0.23, 2.51) at ages ≤5 and at 0.67 units (95% CI: 0.05,1.30) at ages ≤14. In these models, parental imprisonment for female offspring at ages ≤5 is significantly associated with a BMI gain of 2.69 units (95% CI: 0.92, 4.46), increased SBP of 5.89 mmHG (95% CI: 1.18, 10.53) and DBP of 3.79 mmHG (95% CI: 0.34, 7.23). Female parental imprisonment at age ≤14 is also associated with an increased BMI of 1.07 units (95% CI: 0.09, 2.06). No significant associations are found for male respondents.

**Table 3:** Multivariate growth curve model coefficients and 95% confidence intervals for BMI, SBP, and DBP.

		Body Mass Index	Systolic Blood Pressure	Diastolic Blood Pressure
Parental imprisonment, ≤5 years				
Pooled Sex	Coef.	1.37*	2.89	2.34
	95% CI	0.23, 2.51	-0.55, 6.24	-0.07, 4.76
Males, only	Coef.	-0.03	-0.41	0.79
	95% CI	-1.46, 1.40	-5.35, 4.52	-2.59, 4.17
Females, Only	Coef.	2.69**	5.85*	3.79*
	95% CI	0.92, 4.46	1.18, 10.53	0.34, 7.23
Parental imprisonment, ≤14 years				
Pooled Sex	Coef.	0.67*	0.75	1.05
	95% CI	0.05, 1.30	-1.13, 2.62	-0.28, 2.39
Males, only	Coef.	0.28	-0.48	0.68
	95% CI	-0.50, 1.06	-3.18, 2.21	-1.17, 2.53
Females, Only	Coef.	1.07*	2.01	1.41
	95% CI	0.09, 2.06	-0.59, 4.62	-0.50, 3.32

Notes: Significance: *p<0.05 **p<0.01. Abbreviations: Coef.=Unstandardized Beta coefficient, 95% CI=95% Confidence Interval, BMI=Body Mass Index, SBP=Systolic Blood Pressure, DBP=Diastolic Blood Pressure. BMI models contain measures at ages 14, 21, & 30, while blood pressure measures contain measures at ages 21 and 30. All models contain controls for non-European ethnicity, maternal education, age, and child birth weight. Pooled models contain controls for child sex at birth; pooled models and female-only models contain measures for pregnancy status of female respondents at time of interview

**Fig. 2:**
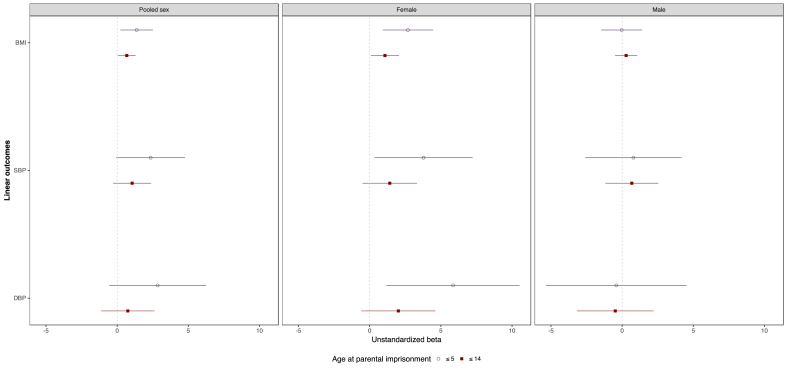
Estimated growth-curve regression coefficients and 95% confidence intervals for outcomes associated with parental imprisonment at ages ≤5 and ages ≤14, by respondent sex. Notes: Plotted results are based on individual growth curve modelling presented in Table 3. Abbreviations: BMI=Body Mass Index, SBP=Systolic Blood Pressure, DBP = Diastolic Blood Pressure. ≤5 denotes parental imprisonment prior to age 5. ≤14 denotes parental imprisonment reported by mothers prior to age 14.

These longitudinal findings are depicted in Fig. 2, showing a pattern of significant associations for the pooled sex and female offspring, but no significant associations for males. In particular, early experiences of parental imprisonment by females are linked with increases in BMI, SBP, and DBP through early adulthood. The development of increased BMI and blood pressure in adolescence or early adulthood are important for indicating potential risk of cardiometabolic diseases in later life.

## 4 Discussion

Using a prospective, longitudinal Australian cohort, the present study finds that parental imprisonment in childhood and adolescence is associated with increased BMI among female offspring in adolescence and adulthood using both cross-sectional and longitudinal analyses. For females experiencing parental imprisonment in the first five years of life, a pattern of BMI-related risks is observed. Parental imprisonment is also associated with increased systolic and DBP in both cross-sectional and longitudinal models, and also with increased waist circumference, increased odds of a waist circumference ≥88 cm, and increased sedentary behavior at age 30. No association between parental imprisonment and health outcomes was observed for men. These findings indicate that parental imprisonment is linked with subsequent increased risk for cardiometabolic diseases in females (but not males) who have experienced parental imprisonment. Parental imprisonment is also consistently linked in single-wave and time-varying models to increased cardiometabolic risk, suggesting results are not due to single-wave attrition in the sample.

These findings are consistent with prior research (1) linking parental imprisonment with obesity and increasing BMI gain in females and (2) associations between familial imprisonment and cardiometabolic health risks among females in middle age (Lee et al., 2014; Roettger & Boardman, 2012; Turney, 2014). While cardiovascular risk in females is normally delayed 5–10 years compared to men and associated with the onset of menopause (Maas & Appelman, 2010), our findings suggest that parental imprisonment may lead to earlier onset of cardiometabolic risk in females compared to females in the general population. It is important to contextualize cardiometabolic risks related to life events later in the life course. For example, research has also found that imprisonment of females is more likely to lead to significantly increased weight gain compared to men, particularly among females who may already be overweight or obese at the time of imprisonment (Gebremariam et al., 2018; Lagarrigue et al., 2017). Given that parental imprisonment in females is linked with increased risk of antisocial behavior and imprisonment (Swisher & Roettger, 2012; Wakefield & Wildeman, 2013), screening for parental and other family-member incarceration, along with incarceration histories, may assist in identifying females at heightened risk for early or increased cardiometabolic risks in adulthood due to these dual risk factors. Findings which identify parental imprisonment as a risk factor for BMI, blood pressure, and related outcomes among females in young adulthood is also critical for designing interventions and policies to reduce health inequalities in later life, particularly since risk factors such as high BMI are linked to early onset of cardiometabolic diseases in later adulthood (Wu et al., 2020).

While small sample size precludes a more detailed analysis of causes and mediators linking parental imprisonment with cardiometabolic risk, it is important to consider a range of contextual factors linked to parental imprisonment. Parental imprisonment is commonly listed as an adverse childhood experience linked to a range of disadvantages in childhood, along with adverse risks that include mental and physical health issues, substance use, antisocial behavior, and poor health behaviors in later life. (Baglivio et al., 2014; Centers for Disease; Control and Prevention, 2010; Heard-Garris et al., 2018; Turney, 2018; Wakefield & Wildeman, 2013). Experiencing multiple childhood adversities, adolescent and adult risk factors, and poor health behaviors are common, potentially accumulating over time in complex ways that may increase cumulative disadvantages in health for children of incarcerated parents as they progress through the life course (Giordano & Copp, 2015; Kinner & Borschmann, 2017; Nurius et al., 2012; Roettger & Dennison, 2018). For example, if we consider our findings linking early childhood imprisonment and BMI at age 30, cumulative risk for cardiometabolic diseases and disability may occur in later life due to associated risks from sedentary behaviors, poor health care access, behavioral health risks such as smoking and heavy drinking, and offspring facing the stress of experiencing a child or family member incarcerated at midlife (Goldman, 2019; Heard-Garris et al., 2018; Lee et al., 2014; Pruchno & Wilson-Genderson, 2015). By exploring these co-occurring risks, future research may help to illuminate how these factors may jointly contribute to the potential burden of disease and disability among those experiencing parental imprisonment as they progress through later stages of the life course.

While administrative and survey data have been widely used to study parental imprisonment and health, the rarity of cohort studies capturing prospective parental imprisonment and child outcomes, including health, are major constraints of existing research (Thornberry, 2009; Wildeman et al., 2018; Wildeman & Lee, 2021). Our use of prospective, longitudinal data from an Australian cohort linked with measures associated with potential cardiometabolic risk factors provides a unique, complementary set of findings compared to US-based results set in the context of mass incarceration (including from cross-sectional research and longitudinal data with retrospective child measures of parental imprisonment). Along with quasi-experimental designs, examining prospective data capturing both parental imprisonment and later health outcomes is critical for future research to identify if parental imprisonment is linked with cardiovascular, metabolic, and other chronic diseases for children in later life. Intensive longitudinal studies containing timing data for parental imprisonment and childhood adversities from early childhood to midlife, either through administrative or cohort data, can also identify potential causal mechanisms.

As recent findings suggest, the timing of parental imprisonment may also constitute an important risk factor for experiencing later adversity (Branigan & Wildeman, 2019; Turney, 2021). Our findings suggest that measurement of parental imprisonment in early childhood may be more strongly linked with cardiometabolic health issues for females than might occur in adolescence and adulthood, while other research suggests that physical disability may be of risk to men (van de Weijer et al., 2021). It is critical for future research to consider the timing of parental imprisonment as a risk factor for health in later life, including potential variations in health risks that may differ by biological sex, age of parental imprisonment, and exposure to related childhood trauma and adversity.

### 4.1 Limitations

While this study has several strengths, it is important to consider these findings in the context of notable limitations. The MUSP clinical data, particularly at age 30, are a small percentage of the original sample and weaken confidence in drawing causal inference from these findings; however, earlier research from the MUSP has found that this attrition does not significantly bias results in analysis (Saiepour et al., 2019). While our results are consistent with prior research on parental imprisonment and BMI, there are a relatively small number of respondents who have mother-reports of parent and partner imprisonment, particularly at ages ≤5, potentially increasing the risk of both type I and type II errors. Data for parental imprisonment are not able to look at imprisonment categorically (e.g., ages 0–5, 6–14) to better examine causality. The small number of respondents experiencing parental imprisonment also limits our ability to explore potential mediators for parental imprisonment, such as risk of poverty, parental substance use, abuse/neglect, parental and offspring mental health, and residential instability identified in existing research (Giordano et al., 2019; Roettger & Houle, 2021; Turney, 2014). The small number of individuals with histories of incarceration also means we are unable to distinguish between biological and stepfather imprisonment, which may yield differing results, particularly in cases where the stepfather's imprisonment may date prior to contact with the biological parent.

As with all longitudinal research, results may also vary due to sociohistorical contexts, such as due to increased imprisonment rates in Australia, among offspring in other countries, or among subpopulations where mass imprisonment may occur in subpopulations over generations. For example, the prevalence of parental imprisonment in Australia varies significantly between Indigenous and non-Indigenous populations (Dennison et al., 2013), which we were unable to test due to the small percentage (<5%) of Indigenous children in the sample and in the general Brisbane population at the time of initial data collection. As noted by Leigh (2020), Australian imprisonment rates risen substantially in the last two decades, though rates are compatible with other Commonwealth countries like the U.K. and Canada. Additional research may help to better establish the factors which may lead to an association between early parental imprisonment and increased adult cardiometabolic risk in females. Social programs, such as universal health coverage and increased social support programs, may also impact these associations, but are beyond the scope of the present study.

## 5 Conclusion

Using a prospective cohort of Australian children, the present study shows that childhood parental imprisonment is associated with increased BMI in females during adolescence and early adulthood. These results hold in both cross-sectional and longitudinal growth curve models. Parental imprisonment experienced by female offspring in early childhood (≤5 years) was associated with increased BMI, SBP and DBP, sedentary behaviors, and increased waist circumference and odds of a waist circumference ≥88 cm. These findings suggest that policies and interventions addressing health risks associated with parental imprisonment may reduce cardiometabolic disease risks in later life.

## Financial conflict

None for all authors.

## Human ethics research review

This project uses anonymized and de-identified secondary data previously collected by the University of Queensland, making it exempt from oversight by the Human Ethics Research Committee at The Australia National University. Original data collects were approved by the appropriate human ethics research committees at the Mater Hospital and The University of Queensland in Brisbane, Australia.

## CRediT authorship contribution statement

**Michael E. Roettger:** Conceptualization, Methodology, Formal analysis, Writing – original draft and revisions. **Brian Houle:** Conceptualization, Methodology, Visualization, Writing – review & editing. **Jake Najman:** Conceptualization, Investigation, Writing – review & editing. **Tara R. McGee:** Writing – review & editing.

## Declaration of competing interest

None for all authors.
